# Determination of tobramycin in eye drops with an open-source hardware ion mobility spectrometer

**DOI:** 10.1007/s00216-022-04050-2

**Published:** 2022-04-05

**Authors:** Nattapong Chantipmanee, Peter C. Hauser

**Affiliations:** Department of Chemistry, University of Basel, Klingelbergstrasse 80, 4056 Basel, Switzerland

**Keywords:** Ion mobility spectrometry, Electrospray ionization, Open-source hardware (OSH), Tobramycin, Eye drops

## Abstract

The analysis of tobramycin was demonstrated successfully as an example for electrospray ionization on an open-source hardware ion mobility spectrometer. This instrument was assembled inexpensively in-house, and required only very few purpose-made components. The quantitative determination of tobramycin required 20 s for a reading. The calibration curve for the range from 50 to 200 μM was found to be linear with a correlation coefficient of *r* = 0.9994. A good reproducibility was obtained (3% relative standard deviation) and the limit of detection was determined as 8 μM. As the concentration of the active ingredient in the eye drops (ophthalmic solutions) is too high for the sensitivity of the instrument, the samples had to be diluted appropriately.

**Graphical abstract:**

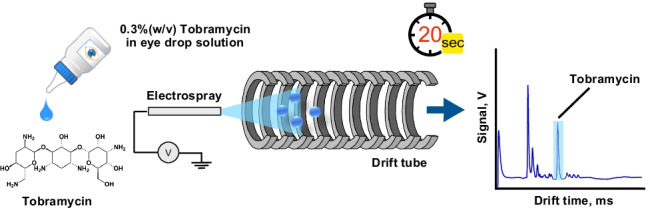

## Introduction

Ion mobility spectrometry (IMS) is an alternative to mass spectrometry, or the separation methods of chromatography or electrophoresis, where ionic species are separated in the gas phase by application of an electric field. In contrast to chromatographic or electrophoretic techniques, the gas-phase separation is much faster. The mobilities of the ions in the inert drift gas are dependent on the collision cross sections of the species, which are largely determined by their size.

A drift tube IMS instrument essentially consists of an ionization source, an ion shutter, a drift tube for separation, and a Faraday detector. The separation is based on the different mobilities of the analyte ions in the electric field in a drift gas (usually nitrogen) due to their different collision cross sections. An IMS instrument is substantially simpler in construction than a mass-spectrometer as it can be operated at ambient pressure and does not require the application of modulated electric fields. It also does not need the high-pressure pumps and manifolds of a high-performance liquid chromatography (HPLC) system. In similarity to CE [1], it is therefore possible to build IMS instruments with limited resources and inexpensively, making the analytical technique available to parties who cannot afford state-of-the-art commercial mass-spectrometers or HPLC instruments. Recently, detailed designs of an instrument [2] and the electronic pulser circuitry [3], required for ion injection, have been published by Clowers and coworkers in the spirit of open-source hardware (OSH), further facilitating in-house construction. We have built such an instrument in our laboratory with some modifications concerning mainly the electronic data acquisition. The electrodes and electrode spacers required for the drift tube could be ordered from a supply house for printed circuit boards, and the shutter grids for the ion injector from a company specializing in micromachining. The electronics for sequencing and data acquisition were based on commercially available units, so that only a few mechanical and electronic parts were needed to be made in-house. The total cost was approximately 5000 Swiss Francs.

Commercially available IMS instruments usually employ a radioactive beta-emitter for analyte ionization of gaseous analytes [4]. However, alternative ionization methods are possible in the form of corona discharges [5, 6], UV-ionization [7, 8], or low-temperature plasmas [9–11]. Electrospray ionization, as a further option, has the advantage of being suitable for liquid samples containing non-volatile species [2, 12]. The study reported herein is a contribution to the exploration of the scope of potential applications of an in-house constructed IMS instrument with electrospray ionization.

Tobramycin is one of the aminoglycoside antibiotics, which is derived from *Streptomyces tenebrarius* [13]. It is widely used to treat Gram-negative infections, particularly against species of *Pseudomonas aeruginosa* [13]. Tobramycin was discovered by Eli Lilly in 1967 [14] and is effective by interrupting ribosomal cell functions [15, 16]. For medical use, tobramycin is typically used in eye drops (ophthalmic solutions) (at 0.3%(w/v)) for treatment of eye infections [17–19]. Like other aminoglycosides, therapy with tobramycin for a long-duration time has potential dose–related side effects of ototoxicity and nephrotoxicity and therapeutic drug monitoring may be applied [13, 15].

Several analytical approaches have been reported for the determination of tobramycin. Immunoassays are one option, but require expensive reagents [20, 21]. For the common separation methods of HPLC, and the alternative capillary electrophoresis (CE), detection by the common UV-absorbance measurement is hampered as tobramycin has no strong chromophore. Chemical derivatization is therefore usually required to enable optical detection by absorbance or fluorescence [15, 22–26]; only when the required sensitivity is low, direct UV-absorbance detection at 210 nm may be employed [27]. Alternatively, evaporative light scattering detection [28, 29], contactless conductivity detection [30, 31], or mass spectrometric detection [32, 33] are employed with HPLC or CE for the determination of tobramycin.

IMS is suitable for analytes which are ionic, or can easily be ionized, and the method has indeed been employed for the determination of pharmaceuticals [34]. Tobramycin can readily be protonated and can therefore be separated and determined as cation. Herein we report on an investigation into the use of the low-cost open-source hardware IMS instrument for the direct analysis of tobramycin in eye drops without requiring derivatization. Electrospray ionization (ESI) was used for ion generation. The detection of other aminoglycosides by ESI-IMS has been reported previously [35], but to the best of our knowledge, this is the first report on the use of the method for the direct quantitative determination of tobramycin in eye drops.

## Materials and methods

### Chemicals

All chemicals were of analytical grade. Tobramycin sulfate (TBM), tetrabutylammonium bromide (T4), and acetic acid were purchased from Sigma-Aldrich (Buchs, Switzerland). Benzalkonium chloride (BAC) was also obtained from Sigma-Aldrich (product no. 12060) and consists of ~ 70% benzyldimethyldodecylammonium chloride and ~ 30% benzyldimethyltetradecylammonium chloride. Methanol (HiPerSolv CHROMANORM) was bought from VWR (VWR Chemicals, Schlieren, Switzerland), and water used throughout the experiment was purified with a Milli-Q system from Millipore (Bedford, MA, USA). All electrosprayed solutions were prepared in 50% (v/v) methanol in water with the addition of 0.01% acetic acid. The diluted samples were filtered through 13-mm syringe filters, Nylon 66, and 0.45 μm (SF1303-2, BGB Analytik, Böckten, Switzerland) before introduction into the ESI-IMS system.

### Ion mobility spectrometer

**Fig. 1:**
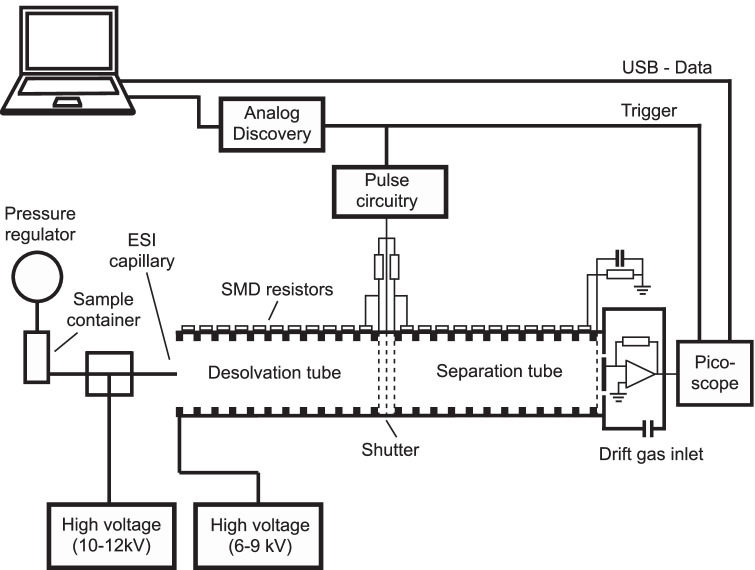
Schematic diagram of the in-house constructed IMS instrument with electrospray ionization

The instrument is based on the design reported by Reineke and Clowers and on our earlier implementation [2, 12]. A schematic diagram of the instrument and its periphery is shown in Fig. 1, and the operating parameters are summarized in Table 1. The IMS tube is divided into two regions, viz. the desolvation and drift zones with 10.6 cm in length each. The drift tube was constructed from stacked ring electrodes (1.6 mm thickness) and spacers (2.0 mm thickness) made from printed circuit board material [2], and these were ordered from PCBway (www.pcbway.com), as well as the two alignment boards fitted with 1-MΩ surface mount resistors (± 1% resistor, Stackpole Electronics, Raleigh, NC, USA) to create the drift field. The high-voltage generator (CZE1000R) was obtained from Spellman (Hauppage, NY, USA). The set of electrodes and spacers were stacked together on four PEEK rods (24.0 cm in length each) through holes at the corners of the PCBs. The injection grids according to the Reineke and Clowers design [2] were obtained from Newcut (Newark, NY, USA). The pulse generator for driving the injection was described by Garcia et al. [3] and purchased from GAA Custom Electronics, Benton City, WA, USA (www.mstar2k.com/gaace-home). Ion detection was carried with a Faraday plate connected to a current-to-voltage converter based on a LMC6001 operational amplifier from Texas Instruments (Dallas, TX, USA) with an amplification of 4.7 × 10^9^ V/A. The signal was recorded by a 16 bit-resolution PC oscilloscope (Picoscope 4262, Picotech, St. Neots, UK). An Analog Discovery 2 unit (Digilent, Pullman, WA, USA) was employed to sequence the injections and trigger the signal acquisition. Nitrogen (N_2_) gas (99.999% purity, PanGas, Pratteln, Switzerland) was employed as the drift gas, and its flow was regulated with a mass-flow controller with a range of 0–1000 mL min^−1^ (Bronkhorst, Aesch, Switzerland).

**Table 1:** Operating parameters

Electrospray ionization	
Pressure (bar)	0.125
Flow rate (μL/min)	2.0
ID of capillary (μm)	50
OD of capillary (μm)	375
Length of capillary (mm)	5.0
Electrospray voltage (kV)	11.0
Injection gate	
Injection time (μs)	250
Gate pulse voltage (V)	50
Distance between grids (μm)	300
Drift tube	
Desolvation length (cm)	10.6
Separation drift length (cm)	10.6
Field strength (V/cm)	425.0
Electrode thickness (mm)	1.6
Spacer gap (mm)	2.0
N_2_ counter gas flow rate (mL/min)	500
Drift tube temperature (°C)	23 ± 2
Drift tube pressure (Torr)	765 ± 5
Ion detection	
Total gain (V/A)	4.7 × 10^9^
Number of averages (times)	200
Analysis time (s)	20

A fused silica capillary of 5 cm length, 50 μm i.d., and 375 μm o.d. (BGB Analytik, Böckten, Switzerland) was employed to create the electrospray. The solutions were pumped by applying compressed nitrogen at a regulated pressure (using a DR-2–1 valve from Clippard, Cincinnati, OH, USA) to a sealed container (LS-BBRES-1ML, LabSmith, Livermore, CA, USA). Connection to the high-voltage generator (CZE1000R, Spellman, Hauppage, NY, USA) was made via a T-piece (T116-203, LabSmith) with a 0.5-mm-diameter platinum wire (Advent, Oxford, UK). Measurements were commenced about 5 s after turning on the electrospray voltage as it was found that this time period was necessary for equilibration of the system before stable signals were obtained.

### Mass spectrometry

The instrument employed was a LCQ Deca 3D ion trap mass-spectrometer (Finnigan MAT, San Jose, CA, USA). The full-scan positive ion mode with low range (200–600 m/*z*) was employed to obtain the mass spectrum using the Tune Plus software vs. 2.0 (Thermo Fisher Scientific, Waltham, MA, USA). A fused silica capillary of 100 μm inner and 365 μm outer diameter (TSP-100365-M-10, BGB), inserted inside a stainless steel tube under high voltage (4.5 kV) and a syringe pump (KDS 100 legacy, KD Scientific, Holliston, MA, USA), was used to create the electrospray. The flow rate was set at 0.2 mL/min.

### Calulations

The reduced mobilities, *K*_0_ (cm^2^ V^−1^ s^−1^), which can be considered to be a normalization of the measured drift times against the instrumental parameters of drift tube length and field strength as well as the temperature and pressure, were calculated according to the following equation [4]:

1 $$K_0=\left(L/t_d\cdot E\right)\cdot\left(p/p_0\right)\cdot\left(T_0/T\right)$$

where *L* is the length (cm) of the drift tube; *E* the field strength (V/cm); *t*_d_ the drift time (s); *p* and *p*_0_ are the drift tube pressure and standard pressure (760 torr), respectively; and *T* and *T*_0_ are the drift tube temperature and standard temperature (273.15 K) respectively.

The resolving powers, *R*_p_, were calculated according to the following equation:

2 $${R}_{\mathrm{p}}=\left({t}_{\mathrm{d}}/{W}_{1/2}\right)$$

where *t*_d_ is the drift time (s) and *W*_1/2_ is the full width at half maximum (FWHM) height.

The linear regression equation with its precision limits was obtained from the raw data by using the function built into Microsoft Excel (Microsoft Corp., Redmond, WA, USA).

## Results and discussion

### Detection of tobramycin

**Fig. 2:**
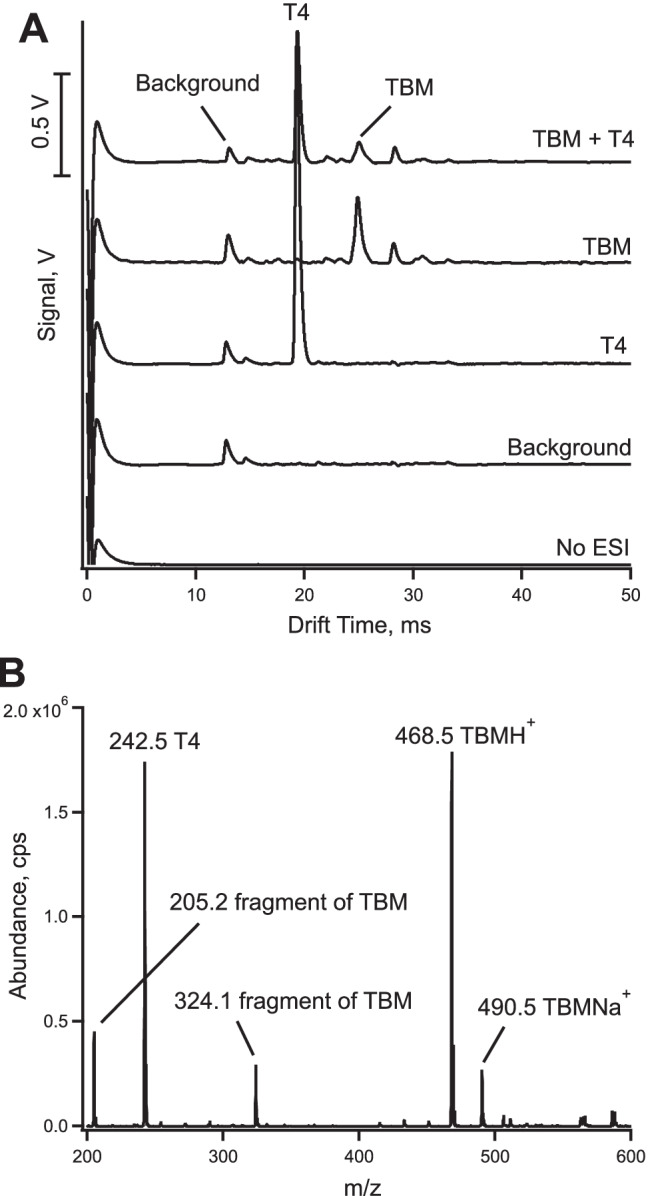
**A** Mobility spectra for tobramycin (TBM)(50 μM), tetrabutylammonium (T4) (5 μM), and a mixture of TBM and T4, obtained with the operating parameters as listed in Table 1. **B** Mass spectrum of the mixture

The detection of tobramycin with the in-house constructed ESI-IMS instrument is illustrated in Fig. 2A. The general operating conditions are summarized in Table 1. A spectrum acquired with the ESI generation turned off is flat except for an early electronic artifact caused by the injection pulse. The background spectrum for the blank electrolyte shows several peaks at drift times between 10 and 20 ms. The solution consists of a 1:1 mixture of methanol and water with the addition of 0.01% acetic acid for protonation of the analyte. The two main peaks in this background spectrum have reduced mobilities (*K*_0_) of 1.90 cm^2^ V^−1^ s^−1^ and of 1.65 cm^2^ V^−1^ s^−1^ and are most likely due to protonated clusters of water [36] with the possible inclusion of methanol. Tetrabutylammonium (T4) was to be used as an internal standard for quantification, and at 5 μM, it gave a single peak at 19.8 ms. This corresponds to a reduced mobility in nitrogen of 1.24 cm^2^ V^−1^ s^−1^, which is very close to the value of 1.25 cm^2^ V^−1^ s^−1^ reported previously by our group for a different instrument [12] and within the range from 1.19 to 1.42 cm^2^ V^−1^ s^−1^ found by Fernandez-Maestre in a literature survey [37]. As can also be seen from Fig. 2A, the injection of tobramycin at 50 μM yielded one main peak at 25.1 ms as well as a series of much weaker peaks at longer drift times, which presumably are adducts. The reduced mobility for the main peak of tobramycin was calculated as 0.97 cm^2^ V^−1^ s^−1^. The last trace of Fig. 2A shows the successful injection of tobramycin together with the internal standard tetrabutylammonium.

In order to confirm the peaks in the ion mobility spectrum, the solution containing the mixture of tobramycin and tetrabutylammonium was also analyzed by electrospray ionization mass spectrometry. As can be seen in Fig. 2B, the main peak for tobramycin is the protonated molecular ion (MH^+^) with its mass-to-charge ratio (*m*/*z*) at 468.5. Some smaller adduct peaks are found at higher masses, with the most prominent one being the sodium adduct (M + Na^+^). This matches the pattern found with IMS. A single peak is found for tetrabutylammonium, again in agreement with the IMS spectrum. The two fragments for tobramycin at 324 and 205 m/*z* units were also reported for mass spectrometry by other groups [32, 33]. Two barely recognizable peaks are present between the T4 peak and the main peak for TBM, which possibly may be due to fragments. However, fragmentation under the IMS conditions is expected to be less pronounced due to the milder working conditions (e.g., working at room temperature).

### Optimization

**Fig. 3:**
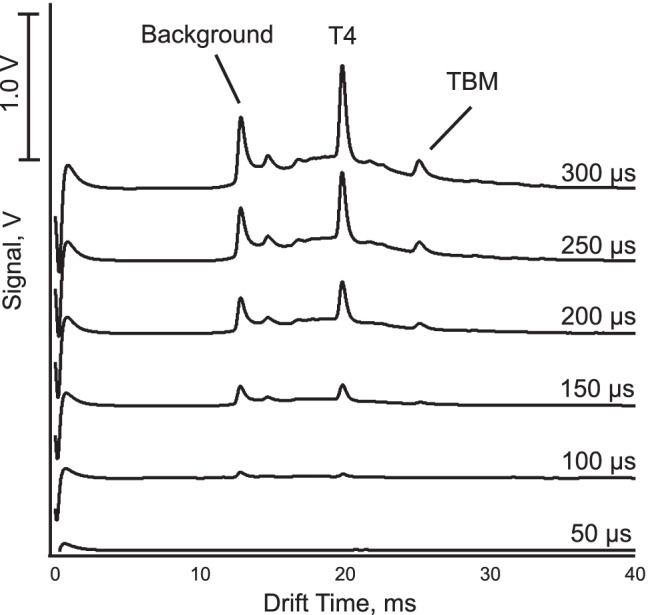
Mobility spectra obtained for different injection times from 50 to 300 μs. The concentrations are shown in Fig. 2, and the operating parameters are listed in Table 1

**Fig. 4:**
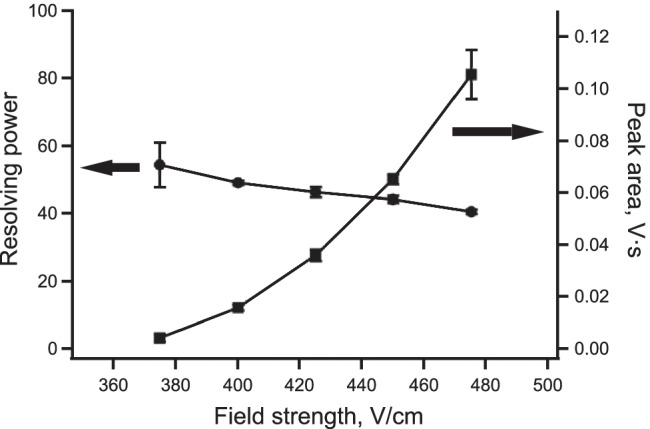
Effect of the electric field strength on the peak area and resolving power for 50 μM TBM with the operating parameters as listed in Table 1. The error bars are the standard deviations for *n* = 3

The effect of the injection time (50–300 μs) for 50 μM of tobramycin (TBM) with tetrabutylammonium (T4) of 5 μM on the mobility spectra was studied. It can be seen in Fig. 3 that when the injection time is increased, as expected, the peak intensity of tobramycin and of T4 is enhanced. However, on the other hand, the resolving power for TBM was found to decrease from 50 to 36 when increasing the injection time from 150 to 300 μs. As a compromise, an injection time of 250 μs was adopted for the subsequent measurements. For further optimization also, the effects of the field strength on the sensitivity and the resolving power were investigated, and the results are given in Fig. 4. The field strength was found to have an effect on the resolving power. This is expected and the theory predicts a maximum, which is depends on the mobility of the species [38]. The reason why here no maximum is found must be due to the also expected strong loss of sensitivity at low field strength [39]. For the low end of the examined field strength range, the signal was indeed very small (leading to poor precision for the lowest data point) and not measurable for lower applied voltages. The use of field strengths at the higher end is therefore desirable as the signal intensity increases strongly, but as the resolving power might be critical, this will not always be possible. However, for the highest applied voltage, a significant loss of precision is also observed.

### Selectivity

**Fig. 5:**
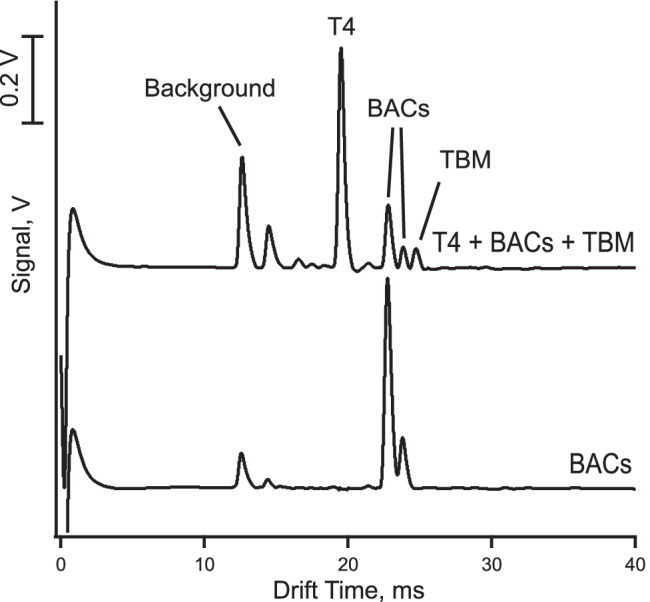
Mobility spectra for the benzalkonium chloride (BACs) cations (5 μM), and a mixture of the BACs (5 μM) with T4 (5 μM), and TBM (50 μM) obtained with the operating parameters as listed in Table 1

The eye drops to be analyzed with the method contain other active ingredients and a number of excipients which are either specified on the packaging or simply referred to as inactives. Of the stated species, only the benzalkonium (BACs) leads to cations and therefore potential interferents. The benzalkonium ions are quaternary amines with an alkyl chain of varying lengths. According to Brignole-Baudouin et al. [40], only the benzyldimethyldodecylammonium and benzyldimethyltetradecylammonium homologues are used in ophthalmic solutions. These are added as chloride salts and act as preservatives. Thus, a mobility spectrum was acquired for a standard mixture of the two BACs in order to ascertain that there is no spectral overlap with tobramycin or the internal standard. As can be seen in Fig. 5, the two benzalkonium cations appear in the mobility spectrum between the peaks for T4 and tobramycin, and therefore, they do not interfere.

### Quantification

A calibration curve was acquired for the concentration range from 50 to 200 μM (5 points, 3 replicates each) of tobramycin by using the internal standardization method to compensate for variations in the production of the electrospray. T4 was used as internal standard and added to a concentration of 5 μM to calibration standards and samples. A linear calibration was obtained for this range (*y* = (0.00304 ± 0.00006)·*x* + (0.00524 ± 0.00764), *y* = ratio of the peak areas, *x* = concentration in μM) with a correlation coefficient, *r*, of 0.9994. The relative standard deviation for measurements at the concentration of 100 μM was found to be 3% by taking 6 measurements. The limit of detection was determined as 7.9 μM (3 × standard deviation). Note that the high end of the calibration curve was limited by a loss of linearity. At the low end, it should be possible to extend the working range by averaging more readings.

### Determination of tobramycin in eye drops

**Fig. 6:**
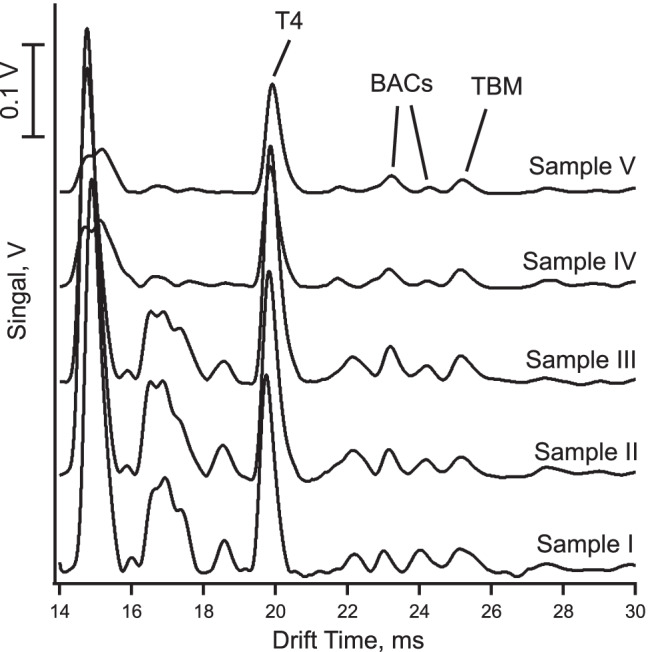
Mobility spectra for the eye drop products obtained with the operating parameters as listed in Table 1

Five commercial eye drops containing tobramycin were bought from a pharmacy in Bangkok, Thailand, and the proposed ESI-IMS method was applied for its determination in these commercial formulations. The samples were diluted by a factor of 125 with the 50% methanol in water (v/v) mixture, containing 0.01% (v/v) acetic acid, to bring the analyte concentrations according to the labels to within the working range of the calibration curves. The stated concentration was 0.3% (w/v) in all cases. T4 was added as internal standard at a concentration of 5 μM. The resulting ion mobility spectra for the eye drop samples are shown in Fig. 6. These spectra also show the internal standard (T4), the benzalkonium peaks, and some unidentified peaks, which may be from excipients subsumed under inactives on the labels. The tobramycin concentrations determined in the products with the internal standardization method are given in Table 2, which are all close to the concentration of 0.3% (w/v) stated on the labels. However, the tobramycin peak for sample I appears to be slightly broadened, which might be due to an overlap with an unknown compound and this might be the reason why the recovery for this sample is slightly high (107%).

**Table 2:** Determination of tobramycin concentration in eye drops

No	Label content %(w/v)	Determined content %(w/v) ± SD *n* = 3
Sample I	0.3	0.32 ± 0.02
Sample II	0.3	0.26 ± 0.01
Sample III	0.3	0.27 ± 0.01
Sample IV	0.3	0.30 ± 0.01
Sample V	0.3	0.29 ± 0.01

## Conclusions

It has been demonstrated that tobramycin used as antibiotics in eye drops can be quantified with a simple IMS device employing electrospray ionization. The method is sensitive and requires only minimum sample preparation and no analyte derivatization. The analysis time is short, even if averaging several readings requiring 20 s each. However, the peak resolution in IMS is generally not as good as that of HPLC or MS, as illustrated by the possible interference observed for one of the samples. ESI-IMS is therefore not suited for complex samples. On the other hand, the application demonstrated serves to illustrate the potential of open-source analytical hardware. The instrument can be built by interested laboratories with little effort.

## Data Availability

Not applicable.

## Code availability

No request from the authors.
